# A retrospective review of how nonconformities are expressed and finalized in external inspections of health-care facilities

**DOI:** 10.1186/s12913-015-1068-9

**Published:** 2015-09-23

**Authors:** Einar Hovlid, Helge Høifødt, Bente Smedbråten, Geir Sverre Braut

**Affiliations:** Institute of Social Science, Sogn og Fjordane University College, PO Box 133, 6851, Sogndal, Norway; The Norwegian Board of Health Supervision, PO Box 8128 Dep, 0032, Oslo, Norway; Stavanger University Hospital, PO Box 8100, 4068, Stavanger, Norway

**Keywords:** External inspection, Quality improvement, Nonconformity, Management system, Clinical governance

## Abstract

**Background:**

External inspections are widely used in health care as a means of improving the quality of care. However, the way external inspections affect the involved organization is poorly understood. A better understanding of these processes is important to improve our understanding of the varying effects of external inspections in different organizations. In turn, this can contribute to the development of more effective ways of conducting inspections. The way the inspecting organization states their grounds for noncompliant behavior and subsequently follows up to enforce the necessary changes can have implications for the inspected organization’s change process. We explore how inspecting organizations express and state their grounds for noncompliant behavior and how they follow up to enforce improvements.

**Methods:**

We conducted a retrospective review, in which we performed a content analysis of the documents from 36 external inspections in Norway. Our analysis was guided by Donabedian’s structure, process, and outcome model.

**Results:**

Deficiencies in the management system in combination with clinical work processes was considered as nonconformity by the inspecting organizations. Two characteristic patterns were identified in the way observations led to a statement of nonconformity: one in which it was clearly demonstrated how deficiencies in the management system could affect clinical processes, and one in which this connection was not demonstrated. Two characteristic patterns were also identified in the way the inspecting organization followed up and finalized their inspection: one in which the inspection was finalized solely based on the documented changes in structural deficiencies addressed in the nonconformity statement, and one based on the documented changes in structural and process deficiencies addressed in the nonconformity statement.

**Conclusion:**

External inspections are performed to improve the quality of care. To accomplish this aim, we suggest that nonconformities should be grounded by observations that clearly demonstrate how deficiencies in the management system might affect the clinical processes, and that the inspection should be finalized based on documented changes in both structural and process deficiencies addressed in the nonconformity statement.

## Background

External inspection systems in which a health-care organization’s performance is assessed according to an externally defined standard are widely used [1]. This activity is a core element in regulatory regimes and in certification and accreditation processes [2, 3]. Assessing a health-care organization’s performance according to external standards has been described by partly overlapping terms such as external inspection, external review, supervision, and audit [4, 5]. In line with Flodgren et al. [6] we will use the term “external inspection” defined by Walshe as, “a system, process or arrangement in which some dimensions or characteristics of a healthcare provider organization and its activities are assessed or analyzed against a framework of ideas, knowledge, or measures derived or developed outside that organization” [7]. The inspection itself is initiated and controlled by an external organization.

The widespread use of external inspections is based on the assumption that they can contribute to improve the quality of care [8]. Previous research has found that external inspections in health care can provide the inspected organizations with useful information for their initiatives and efforts to improve their quality of care [9, 10]. External inspections seem to affect organizational practice, but there is sparse knowledge about how and if such changes in organizational practice lead to improved quality of care [11–13].

The way external inspection systems affect the involved organization is poorly understood [6, 11, 14, 15]. A better understanding of these processes is important to improve our understanding of why effects of external inspections seem to vary between organizations. In turn, this can facilitate development of more effective ways to conduct inspections [6]. It can also help us develop well-designed studies to assess the effects of external inspection systems [12].

One of the core purposes of an external inspection is to contribute to improving quality of care. Quality of care is not a uniquely defined concept. From the patient’s perspective, quality of care is highly dependent on how providers mutually interact to provide high-quality services [16, 17]. Quality of care can thus be considered a property of the health-care system that is dependent on how the services perform as a whole [18]. Accordingly, improving the quality of care is dependent on changing the performance of the system, which in turn implies change in organizational behavior. A basic precondition for external inspections is that the inspected organization is accountable for making necessary changes when nonconformities are encountered. Therefore, the key to improving our understanding of how external inspection might affect the quality of care is to explore how it can facilitate changes in organizational behavior.

Different theoretical frameworks such as institutional theory, public choice theory, and principal agent theory have been suggested as starting points for exploring the effects of regulation and inspection [8]. Our objective is to explore how a core element in a regulatory regime, inspections, can contribute to improve the quality of care. In line with recommendations by Walshe and Boyd [8] we used a framework developed by Hood et al. [19] because it operationalizes the core activities involved in the inspection process. The framework describes three phases of a regulatory regime: direction, detection, and enforcement. Direction refers to actions taken at a system level aiming to affect all the regulated organizations, e.g. developing health-care legislation and national guidelines for delivery of care. Detection refers to actions directed towards individual organizations, e.g. inspecting an organization’s performance in a particular area in relation to standard regulatory requirements. Enforcement comprises actions taken at the individual organizational level to change their performance to comply with the legal requirements, e.g. when the inspecting organization follows up to make sure that necessary changes are implemented when nonconformities are encountered during an inspection. Separating the assessment phase undertaken by an external body from the following enforcement phase where the inspected organization is accountable for implementing necessary changes also complies with the international requirements for inspection standards [20]. For our purpose, only the detection and enforcement phases are relevant because standard setting precedes the actual inspection.

We suggest that elements of these two phases are important to facilitate organizational behavior change. The assessment phase can reveal nonconformities that the inspected organization is obliged to address. During the enforcement phase, the inspecting organization can follow up to make sure that the implemented measures are effective in correcting the nonconformity. The way the inspecting organization states their grounds for noncompliant behavior and subsequently follows up to ensure that the inspected organization implements the necessary changes can have implications for the inspected organization’s change process. We were unable to identify research that specifically addressed how nonconformities are expressed, grounded, and followed up in a health-care setting. The aim of this article is to explore how inspecting organizations in Norway express and state their reasons for nonconforming behavior and how they subsequently follow up the inspected organizations.

### Context

Walshe et al. [5] described proposed approaches to external inspections using the following dimensions: purpose, organization, overall approach, methods, and results. In the following, we will describe inspections in Norway using these dimensions. The Norwegian Board of Health Supervision is a national public institution organized under the Ministry of Health and Care Services that is mandated by the Norwegian Parliament to ensure that health-care and social services are provided in accordance with legal requirements. The Board prioritizes and suggests areas for nationwide inspections based on information about risk and vulnerability. Eighteen county governor offices perform the actual inspections on behalf of the Board.

The standards used for inspections in health-care services are grounded in the legislation laid out in acts and regulations. The legislation is based on two main pillars: health-care services should be safe and effective and provided in accordance with sound professional standards. Furthermore, all organizations that provide health-care services are required to have a management system to ensure that health-care services are provided in accordance with legal requirements. National clinical guidelines provide requirements that are more specific. The rules and regulations that apply for a particular service area are operationalized by the Board into criteria relevant for clinical practice prior to the inspection.

There are two main categories of health-care organizations inspected in Norway: hospitals and municipalities. They provide different kinds of health-care services and represent different types of organizations. The majority of Norwegian hospitals are publicly owned. The hospitals generally represent larger organizations than the municipalities and they provide specialized care. Furthermore, they have a strong hierarchical structure; they are owned by the government and are operated through regional health trusts, which provide a common superstructure. Each municipality represents an independent entity responsible for providing primary health-care services for its inhabitants.

System revision is the main method used to inspect the services in Norway [20]. The inspecting body examines documents, interviews leaders and health-care personnel, and reviews patient records and relevant performance data. After the inspection, an inspection report detailing the findings is delivered to the inspected organization. If the legal requirements are not met, the findings are expressed as a statement of nonconformity. This statement is supported by a number of observations or findings that exemplify why the requirements were not met. The inspecting county governors are also mandated to follow up the inspected organizations until the legal requirements are met.

## Methods

### Design

We conducted a systematic literature review to identify research about how inspecting organizations describe nonconforming behavior and how they follow up the inspected organizations. Flodgren et al. [6] developed a systematic search to identify empirical studies on the effectiveness of external inspections in health care. We used their search strategy as a starting point, modified their search to include qualitative studies, and searched the Cochrane Central Register of Controlled Trials (CENTRAL), Cochrane Database of Systematic Reviews, Database of Abstracts of Reviews of Effectiveness, MEDLINE, EMBASE, CINAHL, Psych INFO, and Web of Science (1980 – December 2014). We did not identify research that specifically addressed how nonconformities in health care are expressed, grounded, and followed up. Therefore, we decided to conduct a qualitative, explorative review.

### Data collection

We used purposive sampling because the objective of our review was explorative [21]. In March 2014, we wrote a letter to the 18 county governor offices in Norway and asked them to send us the correspondence of two recently finalized inspections for review. The two inspections should have encountered nonconformities; one of the inspections should be conducted within specialized care and the other in primary care. We requested the inspection report and the following interchange of documents between the inspection agency and the inspected organization until the inspection was finalized. In the cases where the documentation we received from the county governors was incomplete, we approached them again until our data set was complete. The inspections were carried out during 2011–2013 and finalized during 2013–2014.

### Analysis

Our data consisted of written documents. We imported the documents to Nvivo and performed a content analysis using a combination of a direct and indirect approach, as described by Hsieh and Shannon [22] by combining coding derived from a predefined theoretical framework and codes derived from the data.

In line with the recommendation by Walshe and Boyd [8], we used Donabedian’s [23] structure, process, and outcome model as a theoretical framework to guide our analysis of the content of the nonconformity statements, their corresponding observations, and the inspected organization’s measures to address the nonconformities. Donabedian’s structure, process, and outcome model helps to obtain a better understanding of the preconditions for improving organizational performance and thereby the quality of care. Structure refers to the organizational prerequisites for delivering health services, e.g. buildings, economic and human resources, competence, and infrastructure. The organization’s management system, which is used to control and assure the quality of the services, can be considered part of the structure. Process denotes what is actually done in providing care [23], and outcome is the effect health-care services have on the health status of individuals and populations. Donabedian [23] suggested that structure, process, and outcome are linked through an underlying framework. The quality of the outcomes can thus be understood as a product of the quality of the structural elements and the processes [23]. Accordingly, the quality of the outcomes can be improved by changing the structures, the processes, or a combination of the two [23].

All researchers independently read samples of the documents and thereafter discussed and agreed on an initial coding scheme, which included the nonconformity statement, observations grounding the nonconformity statement, inspecting organization’s follow-up process, measures initiated to correct nonconformity, and finalization of inspection. HH and EH independently coded the documents and added more detailed codes derived from the documents, e.g. specific content of different observations grounding the nonconformity statements and content of measures initiated to correct nonconformities. EH and GSB independently coded the content of the nonconformity statements, the observations, and the measures according to the three domains in our theoretical framework: structure, process, and outcome. Throughout the process of coding, the researchers discussed and compared codes to reach a consensus. Using an iterative process of coding, reflecting on the codes, and condensing, we identified common themes and patterns displaying how the inspecting organization grounded the nonconformities and followed up to ensure that they were dealt with [24].

### Ethical considerations

The protocol of the study was presented to the Norwegian Social Science Data Service, and a formal ethical review was not deemed necessary because all the documents were publicly available and did not contain any kind of personal sensitive information.

## Results

We analyzed the documents of the correspondence between the inspecting and inspected organizations in 36 inspections: 18 from specialized care and 18 from primary care. Based on our analysis we identified the following themes: content of nonconformity statement, content patterns in observations grounding the nonconformity statement, measures to correct nonconformity, and inspection finalization patterns. Table 1 shows the number of inspections for each inspection theme and the frequencies for the patterns we identified. We presented our findings according to the two main phases of the inspection: the assessment and enforcement phases.

**Table 1:** Number of inspection themes and pattern frequencies

	Total number of inspections	Type of care	Number of inspections in which observations do not display how deficiencies in the management system might affect processes	Number of inspections in which observations display how deficiencies in the management system might affect processes	Number of inspections finalized based on changes in structural elements	Number of inspections finalized based on changes in structural and process elements
Child and adolescent psychiatry	10	Specialized	4	6	3	7
Cancer treatment	4	Specialized	1	3	2	2
Stroke treatment	1	Specialized		1		1
Rheumatology	1	Specialized		1		1
Substance abuse	1	Specialized		1		1
Transferal of information to child protection authorities	1	Specialized		1		1
Preventive care for children	7	Primary	4	3	4	3
Compulsory treatment of somatic disorders in patients with cognitive deficiencies	4	Primary	3	1	3	1
Nursing homes and home care	3	Primary	2	1	2	1
Substance abuse	1	Primary	1		1	
Transferal of information to child protection authorities	1	Primary	1		1	
Use of compulsory measures in persons with mental disabilities	1	Primary	1		1	
Handling of medications	1	Primary		1	1	

### Assessment phase

The most commonly used nonconformity statements referred to deficiencies in a combination of structure and process elements in Donabedian’s model. The structural elements of the nonconformity statement typically addressed deficiencies in the inspected organization’s management system, and the process element referred to either clinical processes or a support processes. Generically, it can be expressed in a formula: organization *x* does not have a management system that adequately ensures that process *y* is in accordance with the requirements. The example below illustrates a nonconformity statement for a hospital:

“Hospital A has not established a system for ensuring that patients are assessed and diagnosed according to sound professional practice.”

A small minority of the nonconformity statements solely addressed structural or process elements, e.g. lack of competence or deficiencies in the delivery of a specific health-care service. We did not identify nonconformity statements that addressed outcome elements in Donabedian’s model.

The nonconformity statements were grounded in observations based on data obtained during the inspection, e.g. documents, interviews with personnel and leaders, patient records, or data from the patient administrative system. There was a high degree of coherence between the observations and the nonconformity statement in the sense that they supported and elaborated the grounds for the nonconformity. The observations addressed deficiencies in structure and process elements or a combination of the two. As for the nonconformity statements, the observations concerning structural deficiencies mainly addressed the management system, while deficiencies in process concerned clinical or support processes. Three of the most frequently used themes for observations concerning deficiencies in the management system concerned written guidelines, education, and discrepancy reports.

We identified two patterns characterizing how these observations contributed to support the grounds for the nonconformity statement. In the first pattern, observations describe deficiencies in the management system, but they do not explicitly display or have any reference as to how the deficiencies in the management system might affect the clinical processes. The observations for Municipality A and Hospital B illustrate the first pattern:

Municipality A

“No external or internal education related to nutrition has been undertaken, and the need of competence related to this topic has not been evaluated. The organization has an inadequate system for dealing with discrepancy reports. In the nursing home, there is a file for storing discrepancy reports, but the number of reports is low. Through the interviews, we found that the employees do not get feedback from the management related to how discrepancy reports are handled.”

Hospital B

“The guidelines regarding the referral of patients within the health trust is unclear and inadequate.”

In the second pattern, the observations clearly display how deficiencies in the management system and support processes might affect the clinical processes as illustrated by the following example in which the clinical processes were not conducted in accordance with the national guidelines:

Municipality B

“Preventive health care for children does not routinely perform the activities described in national clinical guidelines. Control at 17–18 months of age is not performed. We did not find any documentation describing why this activity had been omitted. The accountable leader in the municipality has not discovered by means of the management system that the plan for and performance of the health controls are not compatible with national clinical guidelines. Therefore, no activity had been implemented to correct this nonconformity.”

We did not identify observations that addressed the outcome domain in Donabedian’s model. Many of the inspection reports contained a section in which the inspecting organization assessed the inspected organization’s management system. In one of these, we identified a description of the importance of using outcome data to evaluate the organization’s performance:

Hospital C

“It is a major challenge to obtain operational data, e.g. data that can be used to evaluate the effect of provided treatment. It is important that this kind of data is available for managers as well as clinicians so that correct decisions can be made. This kind of data is important for planning as well as evaluation of the services. As it stands now, it may be difficult to evaluate the performance of this department as required by the regulations relating to internal control of the organizations performance.”

### Enforcement phase

In a document accompanying the inspection report, the county governors requested information about how the inspected organization should follow up the nonconformities that had been encountered. The county governors used standardized wording for this request describing the plan of action with time limits for the whole correction process, measures to correct the nonconformities, management’s surveillance of implementation of measures, and management’s assessment of the effect of the implemented measures.

The inspected organization typically responded to this request by developing an overall plan of action with time limits describing when different measures aimed at correcting the nonconformity should be implemented. The most commonly used measures addressed the structural part of the nonconformity statement, e.g. developing or revising written guidelines describing how a specific process should be carried out along with information and education to involved personnel. Other measures that we identified were change in organization and distribution of responsibility, and raising awareness of the importance of filing and following up internal discrepancy reports. The following examples illustrate some of the measures:

Hospital D

“The guideline for dealing with referrals has been approved, and the department has conducted education activities with regard to this guideline. The department has educated all the secretaries regarding the guidelines for managing the waiting lists and how to deal with breaches of maximum acceptable waiting times. Two follow-up courses are planned annually.”

The inspected organization’s initial assessment of the degree of implementation and the effect of the implemented measures was generally vague and relied on qualitative judgments. In many cases, especially in primary care, there was no assessment of effects of measures, but rather a belief that a new written guideline alone would change the practice, as illustrated in the following example:

Municipality C

“The follow up by the managerial team of the municipality to ensure that patient records contain necessary and relevant information is organized in this way. The manager of the section is responsible for elaborating a guideline describing the content of the patient records. This guideline shall be sent to the head of the department of health and social services for approval. When date of revision is reached, the guideline shall be evaluated and sent again for approval by the head of the department of health and social services.”

We identified two distinct patterns as to how the county governors subsequently followed up and finalized the inspections. The first pattern was characterized by the inspection being finalized solely based on documented changes in structural elements addressed in the nonconformity statement. Typically, the inspected organization would revise or develop new written guidelines and provide educational activities for their staff. However, there was no evaluation of whether such changes affected the processes. This pattern is illustrated by the findings from municipality D, which showed nonconformity related to the preparation and dispensing of medication.

Municipality D

“The new guidelines with attached procedures were discussed with leaders on different managerial levels at day seminars to make each leader aware of their responsibilities. The personnel in charge of dispensing medicines from prepared medicine boxes to patients were given internal training according to the procedures established by the municipality. This measure is followed up continuously.”

In the case illustrated above, the county governor finalized the inspection based on documentation of implemented measures aimed at improving structural elements, without documentation of whether the measures affected the processes.

In some of the inspections following this pattern, the county governor requested more documentation of the effect of the measures, but received only qualitative judgments, as illustrated by the following example:

Municipality E

“Finally, we will say that the implemented measures have been shown to be effective. The personnel as well as the managers are more aware about the requirements in chapter 4A of the act regarding the rights of the patients. We have to continue working with documentation in patient records and evaluate to what extent we have made relevant judgments regarding decisions according to chapter 4A.”

A more thorough and meticulous follow up process characterized the second pattern that we identified for finalizing inspections. In cases following this pattern, the county governors did not finalize the inspection until the inspected organization had properly documented that expedient measures had been implemented to correct both the structural and the process elements of the nonconformity statement. This pattern is illustrated by the findings from Hospital E, which showed nonconformity related to the treatment of stroke patients.

Hospital E

“All guidelines will be collected, evaluated, updated, and coordinated with the other hospital in the health trust. We shall establish a system of quality indicators for measuring the result of stroke treatment.”

The county governor requested additional information about the effects of the planned measures, and the quotes below illustrate the key content of the hospital’s answer.

“Using the Global Trigger Tool (GTT) method, we want to analyze five patients every month with the diagnoses I63–I64 and retrospectively evaluate the patient records. In addition, it is worth mentioning that we have had two audits using our adapted GTT method. Both of them showed satisfying follow up related to acute treatment and information letters to the municipal health services. However, we discovered one deviation related to some patients lacking National Institute of Health Stroke Scale (NIHSS) registration in the admission unit.”

In the case illustrated above, the county governor also received documentation about several rounds of patient record reviews using the adapted GTT method, which demonstrated an improvement in process measures. By obtaining process data and reflecting on it, clinicians became aware of undesirable variation, which prompted them to take action to improve their clinical process. Based on this information, the county governor finalized the inspection. This pattern was the most dominant for finalizing inspections carried out in specialized care.

## Discussion

Our main findings relate to how nonconformities are expressed in the assessment phase and followed up in the enforcement phase. We structure our discussion around these two phases.

### Assessment phase

In line with previous research, we found that the nonconformities identified in external inspections addressed deficiencies in the management system, support processes, and clinical processes, but not clinical outcomes [11, 25–27]. The standards used for inspections in our case study are based on requirements in Norwegian legislation, which do not contain specific outcome requirements. Accordingly, we would not expect to encounter nonconformity statements dealing with the outcome domain in our theoretical framework.

The core activity of health-care organizations is to deliver high-quality health-care services in accordance with the legal requirements, and the main purpose of the organization’s management system and corresponding support processes is to ensure that the organization’s clinical processes are in accordance with sound professional standards. We found that many of the observations addressed deficiencies in the management system and the support processes. Power [28] suggested that, “if auditing processes get decoupled from core organizational activities, these effects may be minimal and the audit process becomes an expensive but harmless ritual, which is important for external legitimacy.” Benson et al. [11] found that the corrective measures that addressed deficiencies in support processes had limited effect on patient care, and in line with Power [26], they state that when the feedback concern system processes in general, the intended benefit for the patients should be made clear. We identified two general patterns for how the observations supported the nonconformities: one in which the observations regarding the management system did not explicitly demonstrate how they affected the clinical processes, and one in which they did. Clinicians need an understanding of the reasons for why change is needed [29]. In line with Power [28] and Benson et al. [11], we suggest that observations that clearly demonstrate how deficiencies in the management system and its corresponding support processes affect the clinical processes are more likely to be understandable for clinicians. Consequently, we suggest that it is more likely that such observations can contribute to the implementation of organizational change. This view is also supported by findings from Hilarion et al. [30], who found that using consensus-based indicators as part of external assessment helped to involve professionals in identifying necessary improvement actions.

We did not identify any observations that explicitly addressed the outcome domain in our analytic framework. General comments in a few inspection reports addressed the fact that performance data was not used to assess performance. Access to relevant data about performance, understood as the outcome of the clinical processes, is a key component of a functional management system. Given the fact that most of the nonconformity statements addressed deficiencies in the management system, it is thought provoking that we did not identify any observations addressing the absence of use of performance data to evaluate the outcomes of the clinical processes.

### Enforcement phase

One of the core purposes of an external inspection is to contribute to improvement of the quality of care. According to our analytic framework, improving outcomes is dependent on making changes and improvements in structures and processes. Nonconformities in our review study did generally address a combination of deficiencies in structure and process elements. We identified two patterns for how inspections were followed up and finalized: one pattern in which the inspection was finalized solely based on documented changes in structural deficiencies addressed in the nonconformity statement, and one pattern where changes were documented for both structural and process deficiencies addressed in the nonconformity statement.

When inspections were finalized based on changes in structural deficiencies the inspected organizations typically implemented changes in the management system, e.g. written guidelines and educational activities. Our data do not provide insight into how such measures affected clinical processes and outcomes. Previous research has shown that education and information activities have limited and short-term effects on outcomes [31], and that it takes considerable effort to transform new written guidelines into changes in clinical practice [32]. Therefore, it can be questionable to what extent the most frequently used measures were suited to improve clinical processes and the quality of care. The nonconformities and the supporting observations in our material indicated that the inspected organizations had deficiencies in having a functional management system. This is in line with previous research showing that management systems are not always systematically implemented and that it is questionable to what degree they actually support clinical work [25, 33]. Accordingly, if corrective measures solely address structural deficiencies in the management system, there is an inherent risk that the changes have limited impact on the quality of care.

The second pattern for finalizing inspections was characterized by documented changes in the structural and process deficiencies addressed in the nonconformity statement. Similarly, to the first pattern, the inspected organizations initially implemented measures aimed at correcting the structural deficiencies addressed in the nonconformity statement, e.g. written guidelines. Such guidelines specify how a clinical process should be carried out. In the second pattern, the county governor continued to request an evaluation of the effect of the implemented measures using data that could display to what extent the clinical processes in fact were carried out as specified in the new guidelines.

Access to relevant process data is a basic precondition for quality improvement because they display to what extent improvement efforts are implemented [34]. Process data can make clinicians aware of deficiencies and variations in how the clinical processes are carried out. By requesting process data, the county governor contributed to the shifted focus from merely describing how the clinical process should be carried out to how it actually was carried out. We found that when clinicians reflected on process data, they became aware of variation in the clinical process, e.g. not performing NIHSS registration before treatment. Measuring the process was a prerequisite for becoming aware of variation in processes and implementing measures to eliminate it. Therefore, we suggest that finalizing an inspection based on documented changes in structure and process elements addressed in the nonconformity statement is more likely to contribute to improved quality of care than solely finalizing the inspection based on documented changes in the structure deficiencies addressed in the nonconformity statement.

Each pattern we identified was associated with primary care and specialized care, which might indicate that organizational characteristics are involved in understanding the emergence of these two patterns. Primary care and specialized care represent two different types of organizations in Norway. Hospitals are owned and operated by regional health trusts, which provide a common superstructure. Primary care is provided by municipalities, which are single entities without any formal superstructure. This difference in organizational characteristics can have consequences for the inspecting organization’s ability to follow up inspections. The county governors used the same type of wording to describe the kind of general documentation they requested from both types of organizations following their inspection. However, our findings do not provide insight into the county governors’ actual level of expectations from these two types of organizations. The standards used for inspections in primary care and specialized care are not the same, which leads to different expectations. Our findings do not provide information about how the Norwegian Board of Health Supervision operationalized the requirements prior to the inspections. The way the requirements are operationalized can affect how the county governors conduct and follow up their inspections and contribute to shape their expectations. Based on their longstanding duty of conducting inspections, the county governors possess contextual knowledge about all of the health-care organizations in their region. This knowledge might not be reflected in the written material, but can still contribute to shape their expectations and influence how different organizations are being followed up. We suggest that the two patterns are caused by a complex set of contextual factors related to the inspected organizations themselves, the way the inspections are prepared, and the actions of the inspecting organization during the detection and enforcement phase.

### Limitations and further research

Our findings are based on an explorative review of cases with a limited number of inspections conducted in one country, and should therefore be interpreted with caution. An observational and retrospective study design like ours has limitations of information bias and confounding, and we cannot say whether the patterns we identified are associated with improved clinical processes and outcomes. Written documents were our only source of data. In these documents, we found references to other sources of communication between the county governors and the inspected organizations, e.g. telephone calls and meetings. The county governors’ actions and decisions when following up nonconformities were most likely also based on information not apparent in the written documents. Despite this shortcoming, we assert that the main means of communication during the enforcement phase was based on written documents, and the decision to finalize the inspection was always conveyed to the inspected organization by means of a written document.

We used purposive sampling to conduct our explorative review, and included inspections of primary care and specialized care organizations by all of the county governors in Norway. Furthermore, we included a range of different inspection themes and identified two distinct patterns for how nonconformity statements were supported and enforced. No other themes emerged during the analysis. The frequency counts in Table 1 show numerous cases for each of the patterns that we identified. Therefore, we assert that our data material was sufficiently diverse and rich enough for data saturation [21, 35].

The findings from this exploratory study need to be validated in larger studies. Such research could benefit from a prospective design and by using mixed methods. Future studies should investigate what relevance the patterns we identified for supporting and following up nonconformities have for the inspected organization’s ability to improve their quality of care.

## Conclusion

We identified two patterns for how observations supported the nonconformity statement: one in which it was clearly demonstrated how deficiencies in the management system could affect clinical processes, and one in which this connection was not demonstrated. We identified two patterns characterizing how the inspecting organization followed up the inspected organization and finalized the inspection: one in which the inspection was finalized based solely on documented changes in structural elements addressed in the nonconformity statement, and one based on documented changes in both the structural and process deficiencies addressed in the nonconformity statement. A core purpose of an inspection is that it should contribute to improvement of the quality of care. We suggest that nonconformity statements should be grounded by observations that clearly demonstrate how deficiencies in the management system might affect the clinical processes. Furthermore, inspections should be finalized based on documented changes in both the structural and process deficiencies addressed in the nonconformity statement.
